# Why There Are No Water-Rats in Ireland

**Published:** 1883-06

**Authors:** 


### WHY THERE ARE NO WATER-RATS IN IRELAND.

In an interesting article on the vole or water-rat, by Mr. Grant Allen, in the English *Country Gentleman*, the writer discusses the question why certain animals, such as snakes, vipers, water-rats, etc., are not found in Ireland. For the real solution of the problem, he says, we must go back to the time when England, Ireland and the Continent were united by a broad belt of land across the beds of the English Channel, St. George's Channel, and the North Sea. It is now an ascertained fact that in the very latest geological period, known as the glacial epoch, the whole surface of the British Islands (except an insignificant strip of the south coast) was covered from end to end with a deep coating of glaciers, like that which now envelops all polar lands, and while this condition of things prevailed there were, of course, no animals of any sort in all Britain, or, at any rate, none but a few Arctic types. After the ice melted, however, the existing British fauna, such as it is, began to occupy the land, and the fact that it did so is one proof, though by no means the only proof, that a communication with the Continent then existed across the bed of the North Sea. Now, the animals only pushed their way very slowly into the newly cleared region as the ice melted away, and the consequence is that only some forty kinds of mammals out of the whole European fauna had penetrated as far as England before the gradual submergence of the lowland belt separated it from the Continent by forming the inclosing arms of the sea.

But Ireland lies even further west than England, and there is reason to believe that St. George's Channel had all been flooded some time before the waves of the Atlantic broke down the last link between Dover and Calais. Accordingly, Ireland never got her fair share of land animals at all, for, though the wolf and fox and the Irish hare, and many other quickly migrating creatures had time to cross the intervening belt before the submergence, several smaller or slower creatures, including the vipers, did not get over the ground fast enough, and were thus shut out forever from the Isle of Saints. Among them were the whole race of voles, and that is the reason why Ireland to this day has no water-rats.

---

Bites and Stings.—Apply spirits of hartshorn with a soft rag, if that is not at hand, use soda, saleratus or wood ashes. The venom of bites and stings, being an acid, is neutralized by an alkali.

Scalds and Burns.—Thrust the injured part immediately into cold water; this protects it from the oxygen of the air ; then cover the burn with dry soda or with wheat flour. If severe the patient should live on tea and toast and ripe fruits.

